# Computed Tomography-Based Evaluation of Volume and Position Changes of the Target Region and Organs at Risk During Radiotherapy for Esophageal Cancer: A Pilot Study

**DOI:** 10.3389/fonc.2021.702400

**Published:** 2021-07-28

**Authors:** Yi-mei Liu, Ying-lin Peng, Qi-wen Li, Guanzhu Shen, Ya-ru Ma, Mei-ning Chen, Jun Zhang, Li-rong Fu, Bo Qiu, Hui Liu, Xiao-wu Deng

**Affiliations:** ^1^Department of Radiation Oncology, Sun Yat-sen University Cancer Center, State Key Laboratory of Oncology in South China, Collaborative Innovation Center for Cancer Medicine, Guangdong Key Laboratory of Nasopharyngeal Carcinoma Diagnosis and Therapy, Guangzhou, China; ^2^Nanfang Hospital, Southern Medical University, Guangzhou, China; ^3^Department of Radiation Oncology, The Third Affiliated Hospital of Sun Yat-sen University, Guangzhou, China; ^4^Department of Radiation Oncology, The First Affiliated Hospital of Xiamen University, Xiamen, China

**Keywords:** esophageal cancer, adaptive radiotherapy, target retraction, volume changed ratio, geometric center deviation

## Abstract

**Objective:**

To analyze changes in volume and position of target regions and organs at risk (OARs) during radiotherapy for esophageal cancer patients.

**Methods:**

Overall, 16 esophageal cancer patients who underwent radiotherapy, including 10 cases of intensity-modulated radiation therapy (IMRT) and six of three-dimensional conformal radiotherapy (3D-CRT), were enrolled. The prescription doses for the planning target volumes (PTVs) were as follows: PTV1, 64 Gy/32 fractions; and PTV2, 46 Gy/23 fractions. Repeat computed tomography (CT) was performed for patients after the 5th, 10th, 15th, 20th, and 25th fractions. Delineation of the gross tumor volume (GTV) and OAR volume was determined using five repeat CTs performed by the same physician. The target and OAR volumes and centroid positions were recorded and used to analyze volume change ratio (VCR), center displacement (ΔD), and changes in the distance from the OAR centroid positions to the planned radiotherapy isocenter (distance to isocenter, DTI) during treatment.

**Results:**

No patient showed significant changes in target volume (TV) after the first week of radiotherapy (five fractions). However, TV gradually decreased over the following weeks, with the rate slowing after the fourth week (40 Gy). The comparison of TV from baseline to 40 Gy (20 fractions) showed that average GTVs decreased from 130.7 ± 63.1 cc to 92.1 ± 47.2 cc, with a VCR of −29.21 ± 13.96% (*p*<0.01), while the clinical target volume (CTV1) decreased from 276.7 ± 98.2 cc to 246.7 ± 87.2 cc, with a VCR of −10.34 ± 7.58% (*p*<0.01). As TVs decreased, ΔD increased and DTI decreased. After the fourth week of radiotherapy (40 Gy), centroids of GTV, CTV1, and prophylactic CTV (CTV2) showed average deviations in ΔD of 7.6 ± 4.0, 6.9 ± 3.4, and 6.0 ± 3.0 mm, respectively. The average DTI of the heart decreased by 4.53 mm (from 15.61 ± 2.96 cm to 15.16 ± 2.27 cm).

**Conclusion:**

During radiotherapy for esophageal cancer, Targets and OARs change significantly in volume and position during the 2^nd^–4^th^ weeks. Image-guidance and evaluation of dosimetric changes are recommended for these fractions of treatment to appropriate adjust treatment plans.

## Introduction

Esophageal cancer is a common malignancy in China, and most cases are diagnosed at the middle-to-advanced stages. The main treatment approach is multimodal therapy, involving radiotherapy with other treatments (1, 2). However, because of the relatively large target area of irradiation for esophageal cancer, many normal vital tissues, including the lungs and heart, can be exposed to high radiation doses during conventional radiotherapy. This can cause severe complications, including radiation pneumonitis or late cardiovascular injury, which in turn affects the sufficient supply of radical radiotherapy to the gross tumor volume (GTV) and reduces the patient’s quality of life (3, 4). Recent modern advancements in radiotherapy techniques, including three-dimensional conformal radiotherapy (3D-CRT) and intensity-modulated radiation therapy (IMRT), have enabled the precise design and accurate irradiation of the GTV. These techniques ensure that the GTV receives the required dose for radical treatment and effectively reduces the radiation dose to surrounding normal tissues, thus improving the efficacy of radiotherapy in esophageal cancer (5–7). However, the application of these precise radiotherapy techniques is associated with many unresolved issues such as positioning errors (owing to poor immobilization and reproducibility in radiotherapy for esophageal cancer), internal movement of the GTV and surrounding vital organs owing to the shrinking tumor and breathing movements during fractionated radiotherapy, and changes in physical density and biological characteristics of patients’ internal organs. All these factors can cause a large variation in therapeutic radiation doses and biological effects from the planned treatment (8, 9). To minimize these discrepancies, frequent feedback data, including imaging, delivered dose distribution, and biological tumor parameters, should be incorporated into the treatment process to enable frequent and necessary adjustments to the treatment plan (10, 11) (i.e., adaptive radiation therapy [ART]). In view of these concerns, we conducted this prospective clinical trial (NCT02653521, ClinicalTrial.gov) wherein esophageal cancer patients underwent weekly computed tomography (CT) during treatment to support subsequent modifications in the ART plan. As a pilot study, we used the repeated CT findings from this trial to analyze the target volume (TV) and position changes from the isocenter and surrounding organs at risk (OARs) to provide a clinical reference for developing ART procedure for esophageal cancer.

## Materials And Methods

### General Clinical Information

This study recruited 16 locally advanced esophageal squamous cell carcinoma patients who received radical concurrent radiotherapy and chemotherapy at our center’s (Sun Yat-sen University Cancer Center) radiotherapy department between January 2016 and August 2017. Among those patients, 10 underwent IMRT and six underwent 3D-CRT. Details of general patient information are described in **Table 1**. The inclusion criteria were as follows (1): histopathologically confirmed esophageal squamous cell carcinoma (2), stage III-IVB (6^th^ edition AJCC/UICC staging) tumor that was inoperable or where patients refused surgery (3), no history of chest radiotherapy, and (4) Eastern Cooperative Oncology Group score 0-1. This study was approved by the IRB Committee of Guangdong Society for Prevention and Treatment of Thoracic Tumors, with the approval number of 201512002, and all enrolled patients provided written informed consent.

**Table 1:** General patient information.

Number	Sex	Age (years)	TNM staging	Tumor location	Radiotherapy technique	Chemotherapy protocol
Patient 1	Male	62	T4N1M0	Mid-thoracic	IMRT	Docetaxel+nedaplatin
Patient 2	Male	44	T3N1M0	Mid-thoracic	3D-CRT	Docetaxel+cisplatin
Patient 3	Male	75	T4N1M0	Cervical	3D-CRT	Cisplatin+tegafur
Patient 4	Male	62	T2N1M1	Upper thoracic	IMRT	Docetaxel+nedaplatin
Patient 5	Male	60	T4N1M1	Mid-thoracic	IMRT	Docetaxel+nedaplatin
Patient 6	Male	66	T3N1M0	Mid-thoracic	IMRT	Docetaxel+nedaplatin
Patient 7	Male	53	T3N3M1	Mid-thoracic	IMRT	Docetaxel+nedaplatin
Patient 8	Male	54	T4N1M0	Upper thoracic	3D-CRT	Docetaxel+nedaplatin
Patient 9	Male	67	T3N1M0	Upper thoracic	3D-CRT	Cisplatin+tegafur
Patient 10	Male	64	T3N1M0	Upper thoracic	3D-CRT	Docetaxel+nedaplatin
Patient 11	Male	61	T3N1M0	Cervical	3D-CRT	Docetaxel+nedaplatin
Patient 12	Male	56	T4N1M1	Mid-thoracic	IMRT	Docetaxel+nedaplatin
Patient 13	Male	60	T3N1M1	Upper thoracic	IMRT	Docetaxel+nedaplatin
Patient 14	Male	50	T4N1M0	Cervical	IMRT	Docetaxel+nedaplatin
Patient 15	Male	62	T3N1M0	Upper thoracic	IMRT	Docetaxel+nedaplatin
Patient 16	Male	61	T3N1M1	Upper thoracic	IMRT	Docetaxel+nedaplatin

### Image Acquisition

(1) *Planning CT*: Enhance CTs were performed using a wide-bore, 16-row CT simulator positioning system (Brilliance Big Bore, Philips). Patients were immobilized using a vacuum cushion in the supine position. The CT voltage was 140 kV; tube current, 250 mAs; scanning and reconstruction slice thickness, 5 mm; and slice spacing, 3 mm. Planning CT images were imported to the radiotherapy planning system (Monaco, V5.11, ELEKTA AB) by the physician responsible for target delineation and treatment plan design.

(2) *Repeat CTs (once per week)*: After patients provided informed consent, repeat enhance CTs were performed on the day of administering the fifth (10 Gy), 10th (20 Gy), 15th (30 Gy), 20th (40 Gy), and 25th (50 Gy) fractions. Scanning conditions and ranges were the same as those applied for planning CT. The acquired images were imported into the radiotherapy planning system for target and OAR delineation.

### Target and OAR Delineation

Target and OAR delineation was performed by the same physician for both planning and repeat CT images of patients. The GTV included the primary esophageal lesion and positive lymph node zones, while the clinical target volume (CTV1) included the GTV plus a margin of 0.5 cm in the right-left and antero-posterior directions and a margin of 1.5 cm in the superoinferior direction, as well as the positive lymph node stations. Prophylactic CTV (CTV2) included the paraesophageal nodes and mediastinal lymph node stations 2, 4, and 7. CTV2 also included the supraclavicular area for cervical and upper thoracic esophageal cancer patients and the pericardial lymph nodes, left gastric artery, and lesser curvature lymph nodes for lower esophageal cancer patients. OAR delineation included the spinal cord, heart, and lungs.

### Design of the Radiotherapy Treatment Plan

All patients were treated with 3D-CRT (six patients) or IMRT/VMAT (10 patients) technique using 6 MV X-ray. The planning target volumes (PTV1 and PTV2) were formed by adding a margin of 0.5 cm around CTV1 and CTV2, respectively, and the prescription doses were 64 Gy/32 fractions for PTV1 and 46 Gy/23 fractions for PTV2. Treatment plans were designed for two-phase irradiation. When planning the phase one irradiation (Plan A), the PTV2 (PTV1 included) was prescribed to a dose of 46 Gy/23F and optimized using 60% of the dose constraint for the OARs. In the second phase (Plan B), the PTV1 was escalated to 64 Gy with an additional irradiation of 18 Gy/9F, optimized using the final constraint for the OARs based on the plan A. The full course dose distribution was the sum of these two phases and required to meet the following criteria: at least 95% of the PTV received 95% of the prescription dose; meanwhile, the maximum dose of PTV1 did not exceed 69 Gy. Dose constraints for OARs were as follows: lungs V_20Gy_ ≤30%, mean lung dose ≤19 Gy, maximum dose of spinal cord ≤46 Gy, and heart V_30Gy_ ≤40%.

### Analysis of Geometry and Position Changes in Target and OARs During Radiotherapy

*Changes in TV:* Changes in TV during radiotherapy were described using the volume change ratio (VCR).

$$\text{VC}{\text{R}}_{i}=\frac{\text{T}{\text{V}}_{i}-\text{T}{\text{V}}_{\text{plan}}}{\text{T}{\text{V}}_{\text{plan}}}\times 100\%$$

Where TV*_i_* is the size of the TV in the *i* ^th^ repeat CT image (*i* = 1, 2, 3, 4, 5), and TV_plan_ is the size of the corresponding TV in the planning CT image.

(2) *Shift of target center:* Coordinates of the target center were recorded for each repeat CT (X_TVi_, Y_TVi_, Z_TVi_) and planning CT (X_TVp_, Y_TVp_, Z_TVp_) on the treatment planning system, and its three-dimensional displacement was calculated.

$$\begin{array}{l} \Delta {\text{X}}_{\text{TV}i}={\text{X}}_{\text{TV}i}-\;{\text{X}}_{\text{TV}p}\\ \Delta {\text{Y}}_{\text{TV}i}={\text{Y}}_{\text{TV}i}-\;{\text{Y}}_{\text{TV}p}\\ \Delta {\text{Z}}_{\text{TV}i}=\;{\text{Z}}_{\text{TV}i}-\;{\text{Z}}_{\text{TV}p}\\ \Delta D_{i}=\sqrt{{{\Delta X}_{\mathrm{TVi}}}^{2}+{{\Delta Y}_{\mathrm{TVi}}}^{2}+{{\Delta Z}_{\mathrm{TVi}}}^{2}} \end{array}$$

Where X_TV_ *_i_*, ΔY_TV_ *_i_*, and ΔZ_TV_ *_i_* represent changes in the position of the TV on the *i* ^th^ repeat CT in the left-right (LR), superoinferior (SR), and anteroposterior (AP) directions, respectively (*i* = 1, 2, 3, 4, 5).

(3) *Changes in distance to isocenter (DTI):* Changes in the distance from the coordinates for the OAR centroid (X_OAR_ *_i_*, Y_OAR_ *_i_*, Z_OAR_ *_i_*) to the coordinates for the planned isocenter (X_iso_, Y_iso_, Z_iso_) were measured and recorded for each repeat CT, which were then used to calculate three-dimensional DTI changes.

$$\begin{array}{l} \Delta {\text{X}}_{\text{OAR}i}={\text{X}}_{\text{OAR}i}-\;{\text{X}}_{\text{iso}}\\ \Delta Y_{\text{OAR}i}={\text{Y}}_{\text{OAR}i}-\;{\text{Y}}_{iso}\\ \Delta {\text{Z}}_{\text{OAR}i}=\;{\text{Z}}_{\text{OAR}i}-\;{\text{Z}}_{iso}\\ DTI_{i}=\sqrt{{{\Delta X}_{\mathrm{OARi}}}^{2}+{{\Delta Y}_{{\mathrm{OAR}}_{i}}}^{2}+{{\Delta Z}_{\mathrm{OARi}}}^{2}} \end{array}$$

Where ΔX_OAR_ *_i_*, ΔY_OAR_ *_i_*, and ΔZ_OAR_ *_i_* represent the distance from the OAR centroid to the planned isocenter on the *i* ^th^ repeat CT image in the LR, SR, and AP directions, respectively.

### Statistical Analyses

Data processing and analysis were performed using SPSS (version 17.0, IBM Corp, Armonk, NY, USA). Wilcoxon signed-rank test was performed for the measurement data, considered statistically significant with the p values < 0.05.

## Results

### TV Decrease

**Figure 1:**
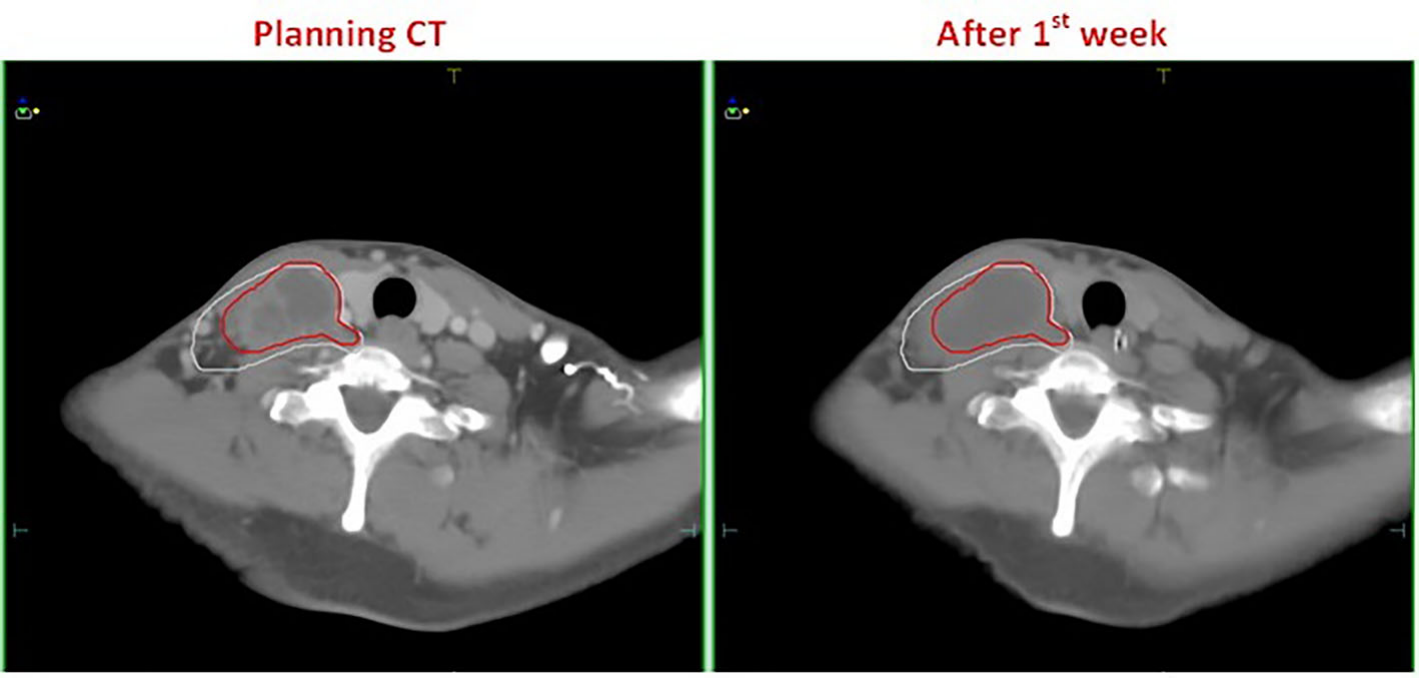
A patient with esophageal cancer who developed edema after the first week of radiotherapy. The left and right figures are the planning CT image and the repeat CT image after the first week of radiotherapy, respectively. The red line is the GTV contour at the planning stage, and the white line is the GTV contour after the first week of radiotherapy.

**Figure 2:**
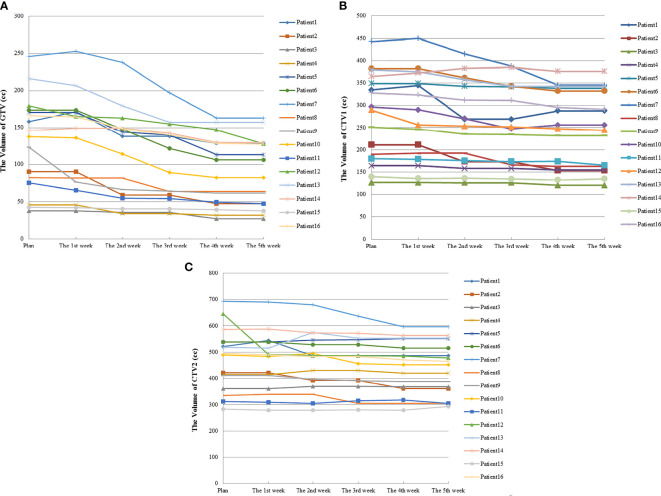
Volume changes in TV during radiotherapy for 16 esophageal cancer patients. **(A)** was the volume changes of GTV; **(B)** was the volume changes of CTV1; **(C)** was the volume changes of CTV2.

Among 16 patients enrolled, none showed a significant decrease in TV after the first week of radiotherapy, and in some cases, TV even increased because of edema (**Figure 1**). After the second week of treatment, significant decreases in GTV and CTV1 were observed, with VCRs of −15.24 ± 12.93% and −5.73 ± 6.47%, respectively. These decreases in TVs continued during the third and fourth weeks of radiotherapy and then began to slow down between the fourth (40 Gy) and fifth weeks, after which changes in TV were not significant. The comparison of TV between the plan and fourth repeat CT showed a decrease in GTV from 130.7 ±63.1 cc to 92.1 ± 47.2 cc (t=−3.516, *p*=0.000), with a VCR of −29.21 ± 13.96% (t=−3.516, *p*=0.000), while CTV1 decreased from 276.7 ± 98.2 cc to 246.7 ± 87.2 cc (t=−3.206, *p*=0.001), with a VCR of −10.34 ± 7.58% (t=−3.464, *p*=0.001). These changes in TV and ratio of decrease at different radiation doses are shown in **Figure 2** and **Table 2**.

**Table 2:** The Volume (cc) and Volume change ratio (VCR) (%) changes of Target.

Target	Plan (0 Gy)	1^st^ week (10 Gy)	2^nd^ week (20 Gy)	3^rd^ week (30 Gy)	4^th^ week (40 Gy)	5^th^ week (50 Gy)
**V_GTV_ (cc)**	130.66± 63.06	126.74 ± 65.00	112.08 ± 60.60^**^	101.86 ± 52.44^***^	92.12 ± 47.22^***^	90.66 ± 46.10^***^
**VCR_GTV_ (%)**	0	−3.45 ± 10.38%	−15.24 ± 2.93%^**^	−21.63 ± 2.33%^**^	−29.21 ± 3.96%^**^	−30.26 ± 11.31%^**^
**V_CTV1_ (cc)**	276.70 ± 98.23	271.45 ± 95.24	260.00 ± 93.92^**^	252.74 ± 90.54^**^	246.75 ± 87.24^**^	245.92 ± 87.37^**^
**VCR_CTV1_ (%)**	0	−0.85 ± 3.28%	−5.73 ± 6.47%^**^	−8.21 ± 6.95%^**^	−10.34 ± 7.58%^**^	−10.66 ± 7.52%^**^
**V_CTV2_ (cc)**	472.87 ± 117.74	462.73 ± 108.60	460.56 ± 109.19	452.10 ± 103.84^*^	444.56 ± 99.69^*^	443.79 ± 99.14^**^
**VCR_CTV2_ (%)**	0	−1.51 ± 6.17%	−2.05 ± 7.32%	−3.74 ± 7.17%	−5.18 ± 7.87%^*^	−5.28 ± 8.15%^*^

All were compared with plan levels.

*P < 0.05, **P < 0.01, ***P < 0.001, using Wilcoxon signed-rank test.

VCR, volume change ratio; V_GTV_, volume of GTV; V_CTV1_, volume of CTV1; V_CTV2_, volume of CTV2.

### Changes in Position of Target Center

**Figure 3:**
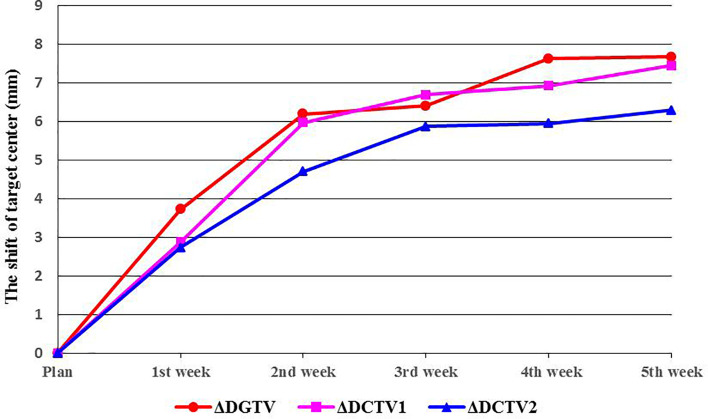
Shifts in target center. ΔDGTV, ΔDCTV1, and ΔDCTV2 represent displacements of the GTV, CTV1, and CTV2 centers, respectively.

As the number of treatments increased, the centroids of the TV (GTV, CTV1, and CTV2) showed greater displacement (ΔD). Compared with the treatment planning stage, significant changes were observed in the position of the target centroid after the first week of radiotherapy. This displacement continued to grow with the increasing number of treatments and was the most significant between the first and second weeks of therapy. By the end of the fourth week (40 Gy), the average displacements for GTV, CTV1, and CTV2 were 7.6 ± 4.0 mm, 6.9 ± 3.4 mm, and 6.0 ± 3.0 mm, respectively, and the displacements were statistically significant (p<0.01). Similarly, the displacement in the target centroid became incrementally smaller, and by the end of the fifth week, the displacement of the target centroid had not changed significantly compared with that at the fourth week (**Table 3**, **Figure 3**).

**Table 3:** Position changes of target centroids (mm).

Displacement of target centroids	Plan	1^st^ week	2^nd^ week	3^rd^ week	4^th^ week	5^th^ week
**ΔD**_GTV_	0	3.7 ± 2.9^*^	6.2 ± 2.9^**^	6.4 ± 3.5^**^	7.6 ± 4.0^**^	7.7 ± 3.5^**^
**ΔD**_CTV1_	0	2.9 ± 2.7^*^	6.0 ± 3.2^**^	6.7 ± 3.2^**^	6.9 ± 3.4^**^	7.5 ± 3.4^**^
**ΔD**_CTV2_	0	2.7 ± 2.3^*^	4.7 ± 2.4^**^	5.9 ± 3.2^**^	6.0 ± 3.0^**^	6.3 ± 2.9^**^

All were compared with plan levels.

*P < 0.01, **P < 0.001, using Wilcoxon signed-rank test.

ΔD_GTV_, displacement of GTV centroids; ΔD_CTV1_, displacement of CTV1 centroids; ΔD_CTV2_, displacement of CTV2 centroids.

### Changes in DTI

**Figure 4:**
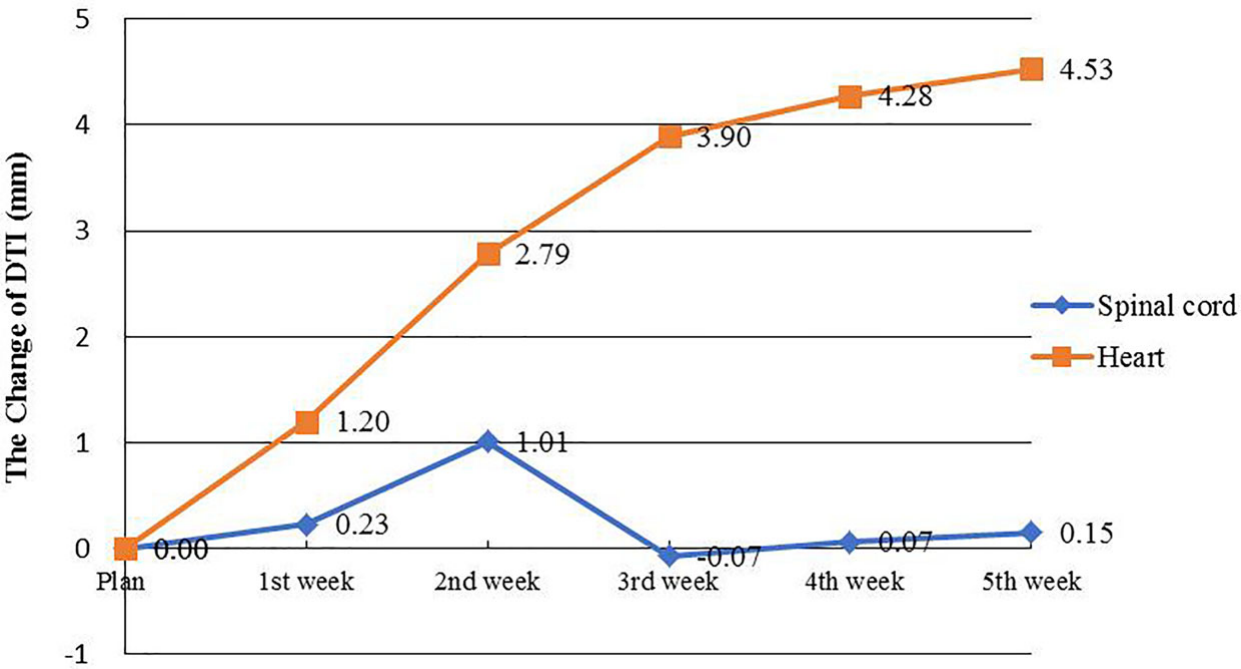
OARs Changes of DTI. The orange line represents the change in average DTI for the spinal cord, and the blue line represents the change in average DTI for the heart.

The DTI did not change significantly as radiotherapy progressed. However, as the number of treatments increased, distance from the centroid of the heart to the planned isocenter significantly decreased. By the end of the fourth week (40 Gy), the average DTI decreased from 15.61 ± 2.96 cm (baseline) to 15.16 ± 2.77 cm (t=−3.104, *p*=0.002), demonstrating an average reduction of 4.53 mm (**Table 4** and **Figure 4**).

**Table 4:** DTI changes under different radiation doses (cm).

Treated Time	0	1 week	2 weeks	3 weeks	4 weeks	5 weeks
(Treated dose)	(0 Gy)	(10 Gy)	(20 Gy)	(30 Gy)	(40 Gy)	(50 Gy)
DTI_heart_	15.61 ± 2.96	15.49 ± 2.82^*^	15.33 ± 2.92^*^	15.22 ± 2.84^*^	15.18 ± 2.77^*^	15.16 ± 2.77^*^
DTI_spinal-cord_	7.22 ± 1.53	7.20 ± 1.56	7.12 ± 1.50	7.23 ± 1.54	7.22 ± 1.56	7.21 ± 1.60

All were compared with plan levels.

*P < 0.01, using Wilcoxon signed-rank test.

DTI_heart_, changes in distance to isocenter of heart; DTI_spinal-cord_, changes in distance to isocenter of spinal-cord.

## Discussion

As radiotherapy technology continues to advance, 3D-CRT and IMRT have gradually become the treatment methods of choice for middle-to-advanced stage esophageal cancer patients. The treatment plans based on these two radiation techniques can provide good conformal dose distributions, which ensures that the target area receives an adequate dose of the radical radiation and effectively reduces the radiation dose to surrounding normal tissues (12). However, during the implementation of radiotherapy, various treatment responses, including a decrease in TV and displacement, as well as changes to the geometric position of surrounding OARs, may occur because the number of treatments and doses increase. This, in turn, may cause treatment errors, with the target area falling into a low-dose area or OARs receiving high-dose radiation. This could result in a failure to achieve expected outcomes and cause serious complications. The use of online image-guided radiation therapy (IGRT) can reduce the effect of such displacement errors to a certain extent (8). However, IGRT can only be used to correct TV center displacement and cannot guarantee the accuracy of dose distribution. Factors such as TV and organ displacement can lead to changes in density distribution, which cause the dose distribution to deviate from the original treatment plan. The use of anatomical imaging at different treatment stages can help inform us about changes in the volume and position of the target area and surrounding OARs, which can then be used to adjust treatment plans and thus implement an ART. In view of this issue, we conducted repeat CTs every five fractions and delineated the target and OAR volume contours on repeat CTs. Based on these data, we analyzed changes in TV, center displacement, and DSI and evaluated their effect on treatment dose distribution to provide support for developing ART for esophageal cancer.

Our findings indicate that during radiotherapy for esophageal cancer, tumor volume generally decreased as the number of treatments increased; however, the response was not significant within the first week of radiotherapy. In fact, after the fifth fraction, three of the 16 patients even showed a slight increase in GTV, from 2.06% to 8.37% (**Figure 2**). Wang et al. (13) reported a similar phenomenon in their esophageal cancer patients, in whom the TV increased rather than decreased during the early stages of radiotherapy. They found that at the 10th fraction, seven of the 38 patients showed an average increase of 22% (4–39%) in GTV. In a study by Britton et al. (14), where 4D-CT was used to evaluate changes in GTV during radiotherapy for non-small cell lung cancer, GTV increased in some patients after the first or second week of treatment. This phenomenon also occurred in other studies involving follow-up imaging evaluation of radiotherapy for intracranial tumors (15–21). Similarly, our study results provided evidence for this phenomenon. This can be attributed to the acute reaction that the esophageal mucosa may have to radiation in some patients during the early stages of radiotherapy, which results in surrounding hyperemia and edema, thereby increasing the GTV evaluated on CT. Furthermore, the accelerated growth and local infiltration of the tumor itself during the early stages of radiotherapy may increase the volume. In addition, we cannot rule out the possibility that this increase may be related to contractions and peristalsis of the esophagus itself or chyme retention at sites of esophageal stricture and obstruction. Although most patients showed no significant changes in tumor volume or deviations in the target center positions after the first week of treatment, this phenomenon should be closely monitored by radiotherapists, and prompt intervention should be initiated as necessary.

Many studies have reported significant changes in TV during radiotherapy for lung cancer, with results mostly suggesting that these occur after 2 weeks of radiotherapy (14, 22–24). In contrast, similar studies on esophageal cancer are relatively scarce. Wang et al. (13) performed repeat CT after 10 and 20 fractions for esophageal cancer patients and found that the average decreases in GTV were 10 and 25%, respectively. This is consistent with our study, in which GTV decreased by an average of 15.24 and 29.21% after 10 (20 Gy) and 20 (40 Gy) fractions, respectively (which was a faster rate than that reported by Wang et al.). In our study, all patients received concurrent radiotherapy and chemotherapy (**Table 1**), and their combined effects may have contributed to the accelerated decrease in GTV in our cohort. After the fourth week of radiotherapy, TV continued to decrease, although at a significantly slower speed. This may be explained by the tumor tissues beginning to undergo fibrosis and therefore having a slower rate of metabolism as treatment progressed, thus diminishing its radiation sensitivity and hence gradually slowing down the shrinkage rate.

Based on our analysis of displacement changes in target centers during radiotherapy for esophageal cancer, we found that the centroid positions of GTV, CTV1, and CTV2 increasingly deviated (ΔD) from their initial positions with increasing number of treatments. After the fourth week of radiotherapy, the average displacements were 7.6, 6.9, and 6.0 mm for GTV, CTV1, and CTV2, respectively, which were slightly larger than those reported in a similar study on radiotherapy for esophageal cancer, which showed that the GTV center for esophageal cancer was displaced by 5.4 mm after 20 fractions (13). This may be because of our application of concurrent radiotherapy and chemotherapy, which resulted in a faster rate of GTV shrinkage. Our study results also confirmed that the DTI decreased as the number of treatments increased and as GTV decreased. This could cause OARs to be closer to high-dose areas of therapeutic radiation, thereby increasing their radiation dose exposure. Our results showed that after the fourth week of radiotherapy (40 Gy), the distance of the heart centroid to the radiation isocenter (DTI_heart_) decreased by an average of 4.53 mm, from 15.61 ± 2.96 cm to 15.16 ± 2.77 cm. For high-dose conformal radiotherapy techniques, including 3D-CRT and IMRT, there was a steep decline in radiation dose to areas around the target region, and a reduced DTI of 4–5 mm was sufficient to cause a large and clinically significant discrepancy in radiation dose. This result also confirmed that during the first 4 weeks of radiotherapy for esophageal cancer, the heart may be exposed to an increasing amount of radiation. Therefore, it is necessary to evaluate the positional deviation of the heart and the resulting dose deviation using online imaging methods. Based on this, the treatment plan should be promptly modified as appropriate to avoid the risk of overexposure of the OARs.

In this pilot study of the ongoing ART trial, the sample size was relatively small and not enough for analyzing the dosimetric changes and modeling the influence on the biological effect like tumor control probability (TCP) or normal tissue complication probability (NTCP). However, this primary result provided useful information to establish appropriate procedure for ART, estimate the necessity, and suitably select the time schedule for replanning the treatment. As the trial goes on and more sample are collected, changes in the volumetric dose (DVH) and their impact on the biological effects to the PTVs and OARs should be further studied.

In conclusion, significant changes in the target volume and position occur during the second and fourth weeks of the radiotherapy for esophageal cancer. The surrounding OARs (especially the heart) may move closer to the planned isocenter as the number of treatments increase. These changes may exacerbate the discrepancy in the dose to the target region and increase the radiation dose to OARs. Therefore, it is recommended to employ online imaging from the second to fourth weeks of radiotherapy for esophageal cancer, to enable the evaluation of changes in target volume and OAR position and their impact on radiation dose and hence determine whether the treatment plan needs to be adjusted, thereby achieving the ART.

## Data Availability Statement

The datasets presented in this study can be found in online repositories. The names of the repository/repositories and accession number(s) can be found below: the Research Data Deposit (Number: RDDB2021001097, https://www.researchdata.org.cn).

## Ethics Statement

The studies involving human participants were reviewed and approved by IRB Committee of Guangdong Society for Prevention and Treatment of Thoracic Tumors. The patients/participants provided their written informed consent to participate in this study.

## Author Contributions

XD and HL contributed to the conception and design of the study. YL, YP, QL, LF, and GS organized the database. YL, YP, YM, BQ, JZ, and MC performed the statistical analysis. YL wrote the first draft of the manuscript. YL and YP wrote sections of the manuscript. All authors contributed to the article and approved the submitted version.

## Funding

This work was jointly supported by Guangdong Esophageal Cancer Institute Science and Technology Program (M201505) and Guangdong Esophageal Cancer Institute Science and Technology Program (Q201908).

## Conflict of Interest

The authors declare that the research was conducted in the absence of any commercial or financial relationships that could be construed as a potential conflict of interest.

## Publisher’s Note

All claims expressed in this article are solely those of the authors and do not necessarily represent those of their affiliated organizations, or those of the publisher, the editors and the reviewers. Any product that may be evaluated in this article, or claim that may be made by its manufacturer, is not guaranteed or endorsed by the publisher.

## Abbreviations

OARs, organs at risk; ART, adaptive radiation therapy; IMRT, intensity-modulated radiation therapy; 3D-CRT, three-dimensional conformal radiotherapy; PTV, planning target volume; GTV, gross tumor volume; VCR, volume change ratio; CTV, clinical target volume; TV, target volume; ESO, esophageal cancer.
